# To Examine Medical Students

**Published:** 1895-10

**Authors:** 


### To Examine Medical Students.

Springfield, Ill., August 31.—The Illinois State Board of Health has issued a call for examinations to be held in Chicago, September 16 and 17, of all matriculates in medical colleges not graduates of some literary institution. The branches used in examinations are reading, writing, grammar, geography, arithmetic, composition, elementary physics and United States history. The questions are furnished by the faculty of the State University, who will rate the papers and issue certificates to successful applicants. These examinations have heretofore been cônducted by the faculty of different medical colleges. The State Board decides that every applicant for matriculation should submit to the same examination conducted by disinterested persons.

